# Coronavirus disease 2019 morbid pulmonary pathology: What did we learn from autopsy examinations?

**Published:** 2021-08-04

**Authors:** Azza Zulfu, Somaya T. Hamid, Khalid A. Elseed, Wadie M. Elmadhoun, Musaab Ahmed, Mohamed H. Ahmed

**Affiliations:** ^1^Department of Pathology, Faculty of Medicine, Omdurman Islamic University, Khartoum, Sudan; ^2^Department of Pathology - University of Medical Sciences and Technology, Khartoum, Sudan; ^3^College of Medicine, Ajman University, Ajman, United Arab Emirates; ^4^Department of Medicine and HIV Metabolic Clinic, Milton Keynes University Hospital NHS, Foundation Trust, Eagelstone, Milton Keynes, Buckinghamshire, UK

**Keywords:** COVID-19, Lung, pulmonary pathology, autopsy

## Abstract

**Background:**

Despite the rapidly expanding data on clinical, epidemiological and radiological aspects of coronavirus disease 2019 (COVID-19), little is known about the disease’s pathological aspects. The scarcity of pathological data on COVID-19 can be explained by the limited autopsy procedures performed on deceased patients.

**Aim:**

This work aims to review and summarize the pulmonary pathological findings observed in COIVD-19 deceased individuals based on recent case series reports published in English up to September 2020.

**Methods:**

A search in Google Scholar, PubMed^Ò^, MEDLINE^Ò^, and Scopus was performed using the keywords “autopsy and COVID-19,” “postmortem and COVID-19,” and “pulmonary/lung pathology and COVID-19.”

**Results:**

Pulmonary autopsy hallmark findings of COVID-19 cases demonstrate the presence of diffuse alveolar damage. The presence of pulmonary thrombi was reported in the majority of patients. Cellular alterations included type 2 pneumocyte hyperplasia, inflammatory cell infiltrates predominantly by lymphocytes, other mononuclear cells, and neutrophils as evident by their specific immunohistochemical markers. Electron microscopy confirmed the presence of virus particles in different cell types, including types 1 and 2 pneumocytes.

**Conclusion:**

The few emerging autopsy reports have substantially contributed towards our understanding of COVID-19 pulmonary histopathological aspects. COVID-19 caused acute severe respiratory manifestations that are the leading cause of morbidity and mortality in infected patients. More studies and research are needed to understand the inflammatory processes and histopathological changes associated with COVID-19 in African populations.

**Relevance for Patients:**

Postmortem investigations advance important mechanistic knowledge on COVID-19 pathophysiology and clinical outcomes and could facilitate provisions for targeted therapies.

## 1. Introduction

Since the outbreak of the coronavirus disease 2019 (COVID-19) pandemic, there is limited data concerning the histopathological aspects of the disease compared to epidemiological, clinical and radiological aspects [1-8]. The scarcity of histopathological reports on COVID-19 is mostly explained by the limited autopsies performed on COVID-19 deceased patients [2]. Despite the global decline in autopsy practice it remains the gold standard for determining histopathological characteristics and associated mechanistic features of newly emerging, re-emerging and unknown diseases [3,5,6,9].

The scarcity of reports on autopsy examination performed on COVID-19 decedents could be explained by the absence of reliable data concerning the degree of COVID-19 infectivity in dead bodies, as most international, regional and national regulatory bodies discouraged the conduct of autopsy, especially in the early days of the pandemic. Unfortunately, there also exists a general unwelcoming attitude of pathologists, clinicians, and society toward autopsy practice [2]. Gradually regulatory bodies released guidelines and safety measure protocols for conducting autopsies in COVID-19 decedents. Consequently, few case reports and case series were published providing insight into the disease’s pathological characteristics [2].

Pathological findings, mainly obtained from autopsies, have greatly contributed towards understanding COVID-19 complexity as a systemic disease that involves multiple organs, with lungs being the most seriously affected [7,10]. Acute severe COVID-19 respiratory disease develops and commonly manifests into severe acute respiratory distress syndrome (ARDS). Histologic examination of COVID-19-associated ARDS autopsy lung tissues revealed an underlying severe form of diffuse alveolar damage (DAD) [7].

COVID-19 induced DAD was characterized by alveolar capillary endothelium, type 2 pneumocyte damage, hyaline membrane formation with a considerable component of fibrin-rich platelets thrombi and microthrombi [7,11,12]. Moreover, recent reports suggested COVID-19 associated pulmonary thromboembolism as a common fatal complication [12]. This article aims to provide a more in-depth understanding of the pulmonary pathological features associated with fatal COVID-19 disease using cell and molecular diagnostic methodologies reported by autopsy case series.

## 2. Methods

A search in Google Scholar, PubMed^Ò^, MEDLINE^Ò^, and Scopus was performed using the keywords “autopsy and COVID-19,” “post-mortem and COVID-19,” and “pulmonary/lung pathology and COVID-19.” The search was restricted to the English language up to September 15, 2020. Ninety-three articles were retrieved. We excluded single case reports, two case reports, commentaries, reviews, editorials, and perspectives. Only case series were included in this review. For cases published in more than one series, we included the most recent publication and excluded the old ones to avoid duplication.

Case series associated with COVID-19 autopsy published between March 2020 and June 2021 were included in this review: Series that reported complete full-body autopsy for major body organs, series that reported whole lung tissues, three series studied tissue through core needle biopsy from selected body organs (lungs, heart, liver, spleen, and lymph nodes), and series that reported on both pulmonary and cardiac tissues. We selected data pertaining only to lung tissue and excluded evaluation of other organ tissues from all series. Furthermore, no clinical or imaging data were included in the study. Reporting was confined to histological features relating to COVID-19 pathogenesis without including features consistent with concomitant lung disease (e.g., bacterial pneumonia, or fungal infections). Histological features related to chronic lung disease or other systemic diseases such as lymphoma were not recorded.

Data were extracted, summarized, and categorized according to the tools used for studying COVID-19 lung tissue pathology within the following sections: (1) Macroscopic findings, (2) microscopic study of lung tissue sections stained by conventional hematoxylin and eosin (H&E) stain and special methods such as immunohistochemistry (IHC), (3) reverse transcription polymerase chain reaction (RT-PCR) assay and RNA *in situ* hybridization (ISH) for severe acute respiratory syndrome coronavirus 2 (SARS-CoV-2) in tissue sections, (4) electron microscopy (EM), and (5) multiplexed measurement of gene expression. For the rest of the review the results and discussion are presented for each section as listed above.

## 3. Results and Discussion

### 3.1. Macroscopic appearance

On macroscopic examination, multiple studies have shown that the lungs of all COVID-19 patients were typically heavy. For instance, Youd and Moore showed the presence of heavy lungs in autopsies in patients (it was noted that the right lung was heavier than the left). No macroscopic thrombo-emboli or areas of infarction were observed [13]. Similarly, Lax *et al*. reported on 11 autopsy cases of COVID-19 in Austria and found that the mean lung weight was 998 g for the right lungs while the mean lung weight was 795 g for the left lungs. Macroscopically, most of the cases (9 out of 11) showed considerable bilateral congestion. Moderate emphysematous changes were seen diffusely in all lungs. Thrombotic material in branches of the pulmonary arteries was observed in all the 11 cases. Pulmonary arterial thrombosis varied in extent from focal to extensive and involved vessels of all sizes. Pulmonary infarctions were present in most cases (9 out of 11) [14]. Besides the increase in weight of the lungs, Wichmann *et al*. showed massive pulmonary embolism in most patients [15]. Studies from the USA reported an increase in lung weights and also the presence of pulmonary emboli in the majority of the COVID-19 patients [16,17]. In African American patients, the same increase in lung weights was noted. The peripheral lung parenchyma featured hemorrhages and firm small thrombi, while the pulmonary arteries at the lung hilum were free of thromboembolism [18]. The increased weight of the lung and edema were also reported from autopsy studies from Germany, Italy, and the USA, with some reports indicating the presence of pulmonary thrombi [19-21].

### 3.2. Histopathological lung findings from COVID-19

Conventional H&E staining of lung tissue sections was a major tool for determining the microscopic histopathological features of postmortem COVID-19 autopsy cases. Special stains were also used to characterize inflammatory cells in examined tissues. Main findings associated with microscopic examinations were the presence of typical features of DAD associated with COVID-19 (this can result from fibrin membrane formation and clumping within alveoli, associated with lymphocyte aggregation, and type 2 pneumocyte hyperplasia and widening of alveolar walls). Some studies demonstrated microthrombi presence while other studies did not show this feature [13-17].

Fox *et al*. reported detailed findings on lung autopsies in ten African American COVID-19 infected decedents. All ten cases showed bilateral DAD (with typical pathological features that typically progress in three phases (i.e., exudation, proliferation, and fibrosis)). The findings of the phases are detailed as follows, exudative phase: Intra-alveolar, proteinaceous edema, inflammation, possible intravascular thrombi, hemorrhage, multinucleated cells, exfoliation, possible or not viral inclusions, proliferative, and fibrosis phases: Interstitial fibrosis, epithelial hyperplasia, emphysema, and remodeling [22].

In addition, the inflammatory cells infiltrate observed in the autopsy tissues was composed of lymphocytes (both CD4+ and CD8+ lymphocytes), mainly located in the interstitial spaces and around larger bronchioles and blood vessels. Besides DAD, fibrin thrombi were also seen in pulmonary circulation in association with the presence of CD61+ megakaryocytes. Importantly, the megakaryocytes were observed within alveolar capillaries and were actively differentiating into platelets. The observed fibrin and platelet aggregates were noted and entrapped inflammatory infiltrate rich in neutrophils [18].

Carsana *et al*. evaluated the lung tissue of 38 COVID-19 autopsy cases from 2 centers in Italy. The features identified in the majority of patients were DAD and fibrin thrombi. There were macrophages in the alveoli and different types of lymphocytes in the interstitium while megakaryocyte numbers were elevated in the lung capillaries [19]. Akermann *et al*. also identified similar features and an increased expression of ACE2-positive lymphocytes (which is absent in the lungs of those without COVID-19) [20].

In agreement with the above studies Bösmüller *et al*. reported similar features related to DAD in COVID-19 infected lungs. Furthermore, an advanced proliferative organized phase in the lungs featured a proliferation of fibroblasts and early collagen fiber deposits within the intra-alveolar exudate. Inflammatory infiltrates were identified in all cases, with one case featuring florid capillary neutrophilic endotheliitis. Microthrombi were seen microscopically in alveolar capillaries and pulmonary microcirculation in all the examined lungs [21]. Similar histological changes were reported by others [22-26]. Table 1 provides a summary of all these studies and their main findings.

**Table 1:** Summary of the 13 COVID-19 case series of autopsy findings

References	Date	Country	No. of cases	Tissue examination tools	Pulmonary findings	Comments
[17]	July 2020	USA	14	Macroscopy, light microscopy (H&E), IHC, EM, qRT-PCR	DAD Thrombosis	Complete autopsy, both macroscopic and microscopic examination for major body organs
[21]	June 2020	Germany	4	Macroscopy, histology (H&E), IHC, EM, RNA studies for SARS-CoV-2 RNA and IL-1β and IL-6 mRNA detection	DAD Thrombosis	Autopsy for lungs only
[18]	May 2020	USA	10	Macroscopy, microscopy (H&E), IHC, RNA labelling EM	DAD Thrombosis	Autopsy examination for lungs and heart
[13]	May 2020	UK	9	Macroscopy, microscopy (H&E)	DAD	Autopsy with microscopic histology for lungs and heart only
[19]	June 2020	Italy	38	Macroscopy, light microscopy (H&E), IHC, EM	DAD Thrombsis	Autopsy for lung tissue only
[14]	May 2020	Austria	11	Macroscopy, microscopy (H&E)+IHC for RT-PCR for SARS-CoV-2 in tissue		Complete autopsy
[15]	May 2020	Germany	12	Macroscopy, microscopy (H&E)+IHC for RT-PCR for SARS-CoV-2 in tissue	DAD Thrombosis	Complete autopsy
[16]	June 2020	USA	7	Autopsy/immunohistochemistry and electron microscopy	DAD Thrombo-embolism	Complete autopsy for major body organs
[23]	September 2020	Iran	7	Microscopy	DAD	Postmortem core needle biopsies from lung, heart, and liver
[24]	March 2020	China	4	Microscopy (H&E), IHC, RT-PCR for SARS-CoV-2 in tissue	DAD	Postmortem Core biopsy for lungs, liver and heart
[20]	May 2020	Germany	7	Macroscopy, histology (H&E/trichrome), IHC, SEM, corrosion casting, direct multiplexed measurement of gene expression	DAD Thrombo- embolism	7- lungs from autopsy of COVID-19 cases compared with 7 lungs from autopsy of (ARDS) cases secondary to influenza A (H1N1) and 10 age-matched, uninfected control lungs
[22]	Aug./Sept. 2020	Italy and USA	6			Only lung tissue studied
[25]	Aug./Sept. 2020	China	3			Core biopsy lung heart liver and LNs

ARDS: Acute respiratory distress syndrome, DAD: Diffuse alveolar damage, COVID-19: Coronavirus disease 2019, SARS-CoV-2: Severe acute respiratory syndrome coronavirus 2, RT-PCR: Reverse transcription polymerase chain reaction, LNs: Lymph nodes, IHC: Immunohistochemistry, H&E: Hematoxylin and eosin, SEM: Scanning electron microscopy, IL-6: Interleukin 6, IL-1β: Interleukin 1 beta

Mansueto summarized the pathological changes as “bilateral interstitial pneumonia with DAD and primary pulmonary hypertension from direct alveolar and endothelial damage. The morphological changes are represented in the first phase by an interstitial inflammatory infiltrate, diffuse vascular congestion, edema and subsequently from proteinaceous edema with or without hyaline membranes lining alveolar walls, inflammatory mononuclear infiltrate with multinucleated giant cells, exfoliation to also intra-alveolar granulocyte inflammation, and position of fibrinoid material which are the true signs of direct damage” [22]. Mansueto *et al*. raised an important observation about the impact of COVID-19 on cardiovascular and neurological systems. For instance, they called for autopsy studies in order to gain understanding about COVID-19 pneumonia, heart failure, myocarditis, coronary syndrome, and importantly about the role of statin and angiotensin-converting enzyme inhibitors. Moreover, Mansueto *et al*. reviewed studies about COVID-19 and the brain. They showed vascular-related and infection-related secondary inflammatory tissue damage to the brain due to an abnormal immune response. They questioned whether these observed brain changes could be attributed to pulmonary changes or cardiovascular changes and what would be the long-term consequence [22,27-30]. We agree with Mansueto *et al*. about the need for more autopsy studies, especially in Africa. Autopsies in Africa may also help in understanding whether ethnic variations may exist in response to COVID-19 infections and the role of malaria in COVID-19.

### 3.3. Nucleic acid testing: RT-PCR assay, RNA ISH and multiplex RNA expression analysis

RT-PCR assay for detecting SARS-CoV-2 (causative agent of COVID-19) in tissue sections was widely used in adjunct with other ancillary methods to demonstrate virion presence in infected pulmonary tissues. Wichmann *et al*. showed that SARS-CoV-2 RNA was detected in the lungs of all 12 infected patients (1.2 × 104-9 × 109 copies/mL) [15]. Bradley *et al*. reported on 14 autopsy cases from Washington state USA, but only tested three cases for SARS-CoV-2 RNA expression. The virus RNA was detected in all three tested lung tissues and was observed in both type 1 and type 2 pneumocytes via EM imaging [17]. Bösmüller *et al*. reported the detection of SARS-CoV-2 RNA by qRT-PCR in all 4 lungs of COVID-19 patients examined in their study [21]. Borczuk *et al*. performed RNA ISH for viral spike RNA, demonstrating reactivity in the tracheal epithelium, areas of hyaline membranes and atypical alveolar type 2 pneumocytes. Virus expression was confirmed by IHC testing of type 2 pneumocyte cells [23]. Tian *et al*. performed RT-PCR for SARS-CoV-2 detection using core needle postmortem lung biopsies. However, they utilized one out of their four-case biopsies due to limited sampling (i.e., insufficient tissue obtained during needle core procedures). The RT-PCR assay for SARS-CoV-2 performed in one lung in one case was positive [25].

Multiplexed RNA profiling was utilized to provide a comprehensive analysis of the host’s pathological processes such as inflammation and angiogenesis due to COVID-19 infection. Akermann *et al*. performed a multiplexed expression analysis of 323 angiogenesis-related genes and 249 inflammatory genes in three groups that included COVID-19, influenza-infected lungs and non-infected lungs. Their study showed that 69 angiogenesis-related genes expression levels were altered in the COVID-19 group compared to non-diseased lungs. Interestingly, both COVID-19 and influenza disease lung specimens showed differential expression of the same 45 angiogenesis-related genes [20], suggesting commonality in the angiogenic pathophysiologic process. Furthermore, 79 inflammatory gene expression levels were altered in COVID-19 compared to controls. Only seven of the differentially expressed inflammatory genes were common between COVID-19 and influenza specimens [20].

### 3.4. EM

Many investigators utilized EM to identify coronavirus-like viral particles of COVID-19 (based on morphology, e.g., size, electron-dense surface, and peripheral spike-like projections) and its localization in infected lung tissue as well as for assessing ultrastructural tissue changes attributed to the virus cytopathic effects. For instance, Rapkiewicz *et al*. identified rare virion presence through EM in COVID-19 infected lung parenchyma [16]. Bradley *et al*. used EM in reporting 14 autopsy cases. Coronavirus particle aggregates were observed in the lungs of five cases. The viral particles were located in the tracheal epithelial cells, the extracellular space and pneumocyte 1 and 2. Indeed, some of the pneumocyte autophagosomes contained viral particles [17]. Fox *et al*. performed EM examination in selected cases from their ten-autopsy series. EM showed particles suggestive of viral infection and ultrastructural tissue changes suggested as a result of viral infection with COVID-19 [18]. Carsana *et al*. utilized EM examination of lung tissue from ten cases out of 38 cases infected with COVID-19. Importantly, particles assumed to be virions were identified by EM in 90% of the autopsies (mainly within cytoplasmic vacuoles within type 1 and type 2 pneumocytes) [19].

Ackermann *et al*. analyzed seven autopsy cases and compared COVID-19 infected lungs with influenza virus-infected lungs and ten uninfected lungs. The lungs in the COVID-19 group revealed a distorted microvascularity with structurally deformed and elongated capillaries and an increase in the intussusceptive pillars (hole like structure), a distinctive feature of intussusceptive angiogenesis (splitting angiogenesis) at which a pre-existing blood vessel splits into two smaller ones. Furthermore, transmission EM of the COVID-19 infected endothelium showed endothelial ultrastructural damage and endothelial intracellular and extracellular space localization of SARS-CoV-2 [20].

Bösmüller *et al*. showed that EM imaging can be used to demonstrate the presence of viral COVID-19 particles within membrane-bound vesicles which were suggested to be endosomes [21]. Similarly, Borczuk *et al*. reported virus particles within the pneumocyte cytoplasm and possibly in endothelial cells. Small blood vessels were noted to show some ultrastructural changes including endothelium cytoplasmic swelling, vacuolization, and basement membrane duplication [23]. It is important to note that EM can be useful but cannot be used by itself. Hence, EM findings need to be supplemented with other diagnostic methodologies.

## 4. Conclusion

Pulmonary autopsy findings associated with COVID-19 cases demonstrate the presence of various types of pathological changes in alveoli, interstitium and blood vessels. DAD, widening of alveolar walls, desquamation, and fibrin clumps with alveolar spaces was the most evident pulmonary pathological changes. In the interstitium, edema, congestion, infarction, and fibrinous exudates were the most common changes. In pulmonary vasculature, thrombo-emboli were the hallmark changes. Pulmonary cellular alterations include type 2 pneumocyte hyperplasia, inflammatory cell infiltrates predominated by lymphocytes, other mononuclear cells and neutrophils as evident by their specific immunohistochemical markers. EM confirms the presence of virus particles in different cell types including types 1 and 2 pneumocytes. The utilization of various molecular techniques and more autopsies can assist in understanding the pathogenesis and clinical outcomes of COVID-19 and hence the provision of targets for therapy.
